# Peripheral Calcifying Epithelial Odontogenic Tumour Mimicking a Gingival Inflammation: A Diagnostic Dilemma

**DOI:** 10.1155/2016/3014892

**Published:** 2016-10-11

**Authors:** Danielle Lima Corrêa de Carvalho, Alan Motta do Canto, Fernanda de Paula Eduardo, Letícia Mello Bezinelli, André Luiz Ferreira Costa, Paulo Henrique Braz-Silva

**Affiliations:** ^1^Department of Stomatology, Division of General Pathology, School of Dentistry, University of São Paulo, São Paulo, SP, Brazil; ^2^Unit of Oral and Maxillofacial Surgery, Santa Casa de São Paulo, School of Medical Sciences, São Paulo, SP, Brazil; ^3^Department of Orthodontics and Radiology, University of São Paulo, São Paulo, SP, Brazil

## Abstract

The calcifying epithelial odontogenic tumour (CEOT) is an extremely rare benign neoplasia, accounting for approximately 1% of all odontogenic tumours. CEOT can have two clinical manifestations: central or intraosseous (94% of the cases) and peripheral or extraosseous (6% of the cases). Although the latter is less common, the peripheral variant has been described as an insidious lesion, since it is usually asymptomatic and may be erroneously mistaken with gingival hyperplasia, hamartomas, or even metastasis of malignant neoplasia. We report a case of a young male patient presenting with a peripheral CEOT in the mandibular posterior region, mimicking a located gingival inflammation.

## 1. Introduction

The calcifying epithelial odontogenic tumour (CEOT) is a rare odontogenic neoplasia characterised by the presence of amyloid material, which can present calcified [1]. This entity, earlier known as “Pindborg Tumour,” has an epithelial origin and accounts for approximately 1 percent of all odontogenic tumours, affecting the mandibular bone in the majority of the cases [1].

Although the intraosseous variant is more common (94% of the cases), studies have shown evidence that this neoplasia affects exclusively soft tissues [1, 2]. Peripheral CEOT is extremely rare and accounts for approximately 13.3 percent of the cases of all peripheral tumours [3]. According to the studies, these tumours derive from epithelial remnants of the dental lamina or from the gingival surface [2].

Differently from the central variant, the peripheral CEOT is more prevalent in females, occurring between the third and fourth decades of life and involving the anterior maxillary region [4, 5]. These tumours are usually asymptomatic and tend to be less aggressive, causing only alterations in soft tissues. They respond to surgical treatment and do not relapse if properly treated [4].

The objective of this study is to describe a case of an uncommon peripheral CEOT in the posterior mandibular region as well as to perform a brief review of the literature.

## 2. Case Presentation

**Figure 1:**
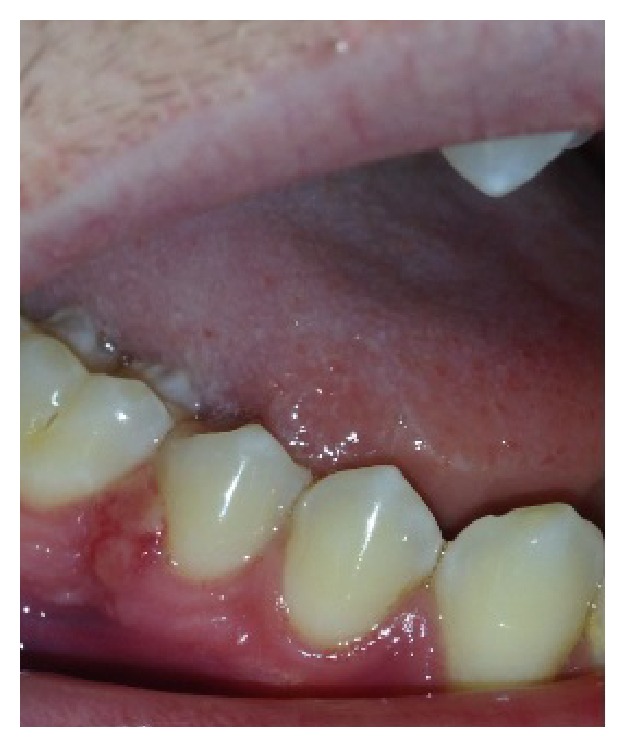
Clinical features of the lesion.

An 18-year-old male patient was referred for evaluation because of an asymptomatic increase in gingival volume, which was lasting for one month. On the clinical exam, the gingiva showed an exophytic lesion with erythematous and irregular surface located in the buccal region, between premolar and first lower molar, and measuring approximately 5 × 5 mm (Figure 1). The patient reported no pain in the region and oral hygiene was regularly performed, with the involved bone region showing no clinical or imagenological alterations. There was no history of local trauma. As medical history, the patient had acute lymphoblastic leukemia, which was successfully treated by means of chemotherapy 1 year earlier than the emergence of the lesion.

**Figure 2:**
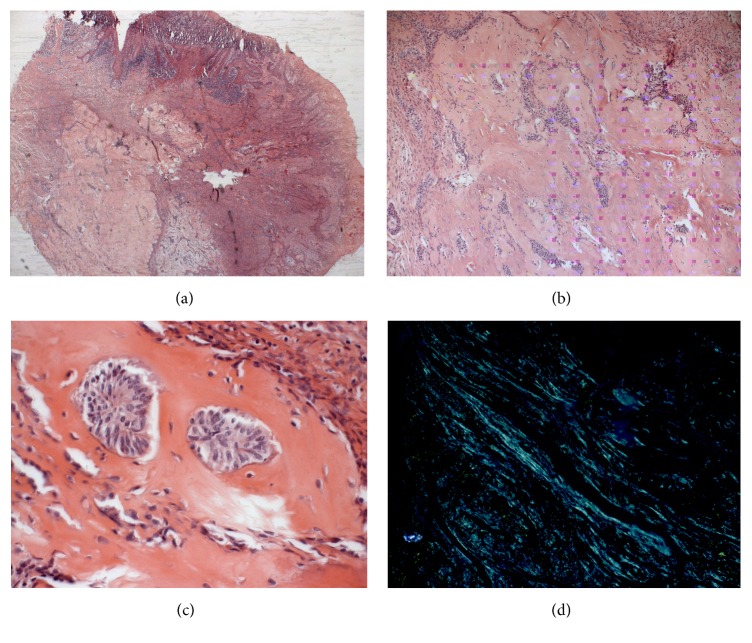
Histopathological features of the peripheral CEOT. (a) A general overview of the lesion, showing the mucosal lining epithelium with areas of ulceration and inflammatory cells infiltration. Proliferation of odontogenic epithelial cells organized in strands, cords, and nests and amyloid-like material (H & E, original magnification: ×25). ((b) and (c)) Strands, cords, and nests of odontogenic epithelial cells dispersed through the amyloid material (H & E, original magnification: ×40 (b), ×100 (c)). (d) Amyloid stained by Congo red showing apple-green birefringence in polarized light (original magnification: ×40).

Considering the lesion's dimensions and aspects suggesting a benign behavior, an excisional biopsy of the region was performed under local anaesthesia. The haematoxylin-eosin stained histological sections showed proliferation of fusiform cells arranged either in bundles or randomly, including intense deposition of amorphous material among them. This substance had an eosinophilic appearance compatible with amyloid deposition associated with strings and islets of odontogenic epithelium dispersed through the neoplasia. The specimen was positive to Congo red under polarized light, showing the amyloid origin of the eosinophilic material. The mucosal lining epithelium showed areas of ulceration and neutrophilic infiltration (Figure 2).

Based on the clinical, radiological, and histopathological characteristics, the case was diagnosed as a peripheral CEOT. The patient was regularly followed up for one year and no sign of relapse was found.

## 3. Discussion

Calcifying epithelial odontogenic tumours are rare neoplasia accounting for a small percentage of all odontogenic tumours. Studies report that there are about 200 cases worldwide [6].

In addition to the rarity of these tumours, the peripheral variant is even more uncommon. Interestingly, 24 related cases were found in the PubMed database [1, 7].

In a recent literature review, Shetty et al. (2014) reported that peripheral CEOT is more common among females in the third and fourth decade of life (mean age of 37.33 years), usually affecting the anterior region of the maxilla [4]. Although the majority of the lesions are unilateral, studies have reported cases of bilateral lesions and tumours in maxilla and mandible simultaneously [2, 8]. However, as well as in our case report, some authors describe a higher rate of this variant among males, affecting areas of canines and premolars in the mandible [1, 2].

With regard to the origin of this neoplasia, it is believed that these tumours only stem from the dental lamina epithelium due to its association with teeth enclosed within the bone. Nevertheless, because of the presence of the gingival variant, other possible origins have been discussed and reported in other studies [4]. As soft tissues are exclusively affected, it has been strongly demonstrated that these tumours can also stem from basal cells of the oral epithelium, which persist in the region following disintegration of epithelial remnants (rests of Serres) [4].

With regard to the differential diagnosis, the peripheral CEOT can resemble clinically or histologically several other lesions such as peripheral odontogenic tumours, odontogenic carcinoma with dentinoid, clear cell odontogenic carcinoma with dentinoid, minor salivary gland tumours, tumour metastasis, reactive hyperplasia, and acute gingival inflammations [9]. According to Shetty et al. (2016), presence of amyloid material, calcifications, absence of mitoses, immunohistochemical positivity to cytokeratin 14, and absence of S-100 protein expression can help to perform a final diagnosis [1]. In addition to these characteristics, the positivity to Congo red under polarized light can be useful for diagnosing and differentiating other lesions [1, 8].

The histopathological aspect of peripheral CEOT is well characterised, consisting of epithelial cells with cytoplasmatic eosinophilic content and amorphous amyloid substance with spots of calcification organized in concentric lamellas [5]. These cells can occasionally have an empty and vacuolated cytoplasm (clear cell variant) and mimic clear cell carcinomas. These cells contain an abundant amount of glycogen and can also have Langerhans cells in some cases. According to the authors, due to higher aggressiveness of the clear cell tumours, CEOTs presenting this cell variation can exhibit greater tissue destruction and higher trend for relapse [10].

Although authors have described the potential reoccurrence of clear cell CEOT [9], studies showed evidence that soft tissue variants are less severe neoplasia as they usually have small sizes (i.e., 0.5 to 2 cm), preserve osseous tissue, and do not tend to relapse if properly removed [1]. Only one case described by Shetty et al. (2014) showed an atypical presentation of peripheral CEOT with great dimensions and calcifications and was treated through maxillectomy [4]. Despite these characteristics in the present case as well as elsewhere, there are a few cases with evidence of relapse [1, 4, 8].

## 4. Conclusion

Due to the above-cited characteristics, the changes in gingival mucosa should be thoroughly examined because of the possibility of development of soft tissue peripheral neoplasia. Moreover, peripheral CEOTs have clinical similarities with several soft tissue lesions and thus their differentiation regarding other pathologies is of extreme importance for adequate treatment and follow-up.
